# High-resolution neutron and X-ray diffraction room-temperature studies of an H-FABP–oleic acid complex: study of the internal water cluster and ligand binding by a transferred multipolar electron-density distribution

**DOI:** 10.1107/S2052252515024161

**Published:** 2016-01-16

**Authors:** E. I. Howard, B. Guillot, M. P. Blakeley, M. Haertlein, M. Moulin, A. Mitschler, A. Cousido-Siah, F. Fadel, W. M. Valsecchi, Takashi Tomizaki, T. Petrova, J. Claudot, A. Podjarny

**Affiliations:** a Department of Integrative Biology, Institut de Génétique et de Biologie Moléculaire et Cellulaire, Centre de Biologie Intégrative, CNRS, INSERM, UdS, 1 rue Laurent Fries, 67404 Illkirch CEDEX, France; b Instituto de Fisica de Liquidos y Sistemas Biologicos, CONICET, UNLP, Calle 59 No. 789, La Plata, Argentina; c CNRS and Université de Lorraine, Laboratoire CRM2, UMR 7036, Vandoeuvre-lès-Nancy, F-54506, France; d Institut Laue–Langevin, 71 avenue des Martyrs, 38000 Grenoble, France; e ILL–EMBL Deuteration Laboratory, Partnership for Structural Biology, 71 avenue des Martyrs, Grenoble 38000, France; f Instituto de Química y Fisicoquímica Biológicas, Universidad de Buenos Aires, Junín 956, C1113AAD, Buenos Aires, Argentina; g Swiss Light Source, Paul Scherrer Institute, 5232 Villigen PSI, Switzerland; h Institute of Mathematical Problems of Biology, Russian Academy of Sciences, Pushchino 142290, Russian Federation

**Keywords:** Neutron protein crystallography, high-resolution room-temperature X-ray crystallography, fatty acid binding protein, protein hydration layer, AIM topological properties

## Abstract

Crystal diffraction data of heart fatty acid binding protein (H-FABP) in complex with oleic acid were measured at room temperature with high-resolution X-ray and neutron protein crystallography (0.98 and 1.90 Å resolution, respectively). These data provided very detailed information about the cluster of water molecules and the bound oleic acid in the H-FABP large internal cavity. The jointly refined X-ray/neutron structure of H-FABP was complemented by a transferred multipolar electron-density distribution using the parameters of the ELMAMII library. The resulting electron density allowed a precise determination of the electrostatic potential in the fatty acid (FA) binding pocket. Bader’s quantum theory of atoms in molecules was then used to study interactions involving the internal water molecules, the FA and the protein. This approach showed H⋯H contacts of the FA with highly conserved hydrophobic residues known to play a role in the stabilization of long-chain FAs in the binding cavity. The determination of water hydrogen (deuterium) positions allowed the analysis of the orientation and electrostatic properties of the water molecules in the very ordered cluster. As a result, a significant alignment of the permanent dipoles of the water molecules with the protein electrostatic field was observed. This can be related to the dielectric properties of hydration layers around proteins, where the shielding of electrostatic interactions depends directly on the rotational degrees of freedom of the water molecules in the interface.

## Abstract 2

Neutron and high-resolution X-ray crystallography were used to determine fully the structure of the internal water cluster in H-FABP. Analysis of the orientation and electrostatic properties of the water molecules showed significant alignment of the permanent dipoles of the water molecules with the protein electrostatic field.

## 1. Introduction   

Water molecules are of the utmost importance for recognition between biological molecules. Many studies, extensively reviewed by Raschke (2006 ▸), have focused on hydration water molecules in protein surfaces, noting that they have slower correlation times than bulk water, in agreement with studies of water molecules in confined spaces. Due to contacts with the confining surfaces, the total number of water–water hydrogen bonds is reduced and their strength is reinforced, increasing the tetrahedrality and lowering the orientational dynamics and therefore the dielectric constant (Gilijamse *et al.*, 2005 ▸). Furthermore, the lack of competing water molecules and the effect of environmental fluctuations in the confined space (Stanley *et al.*, 2009 ▸) lower the diffusion coefficient and increase the viscosity (Chaplin, 2009 ▸). A comprehensive analysis of water in protein interfaces from crystal structures has shown a difference between biological and crystal-packing interfaces; the latter have 50% more water molecules than the former (Rodier *et al.*, 2005 ▸), implying that water molecules are expelled when the biological interactions are completed.

These results imply that the properties of water in hydration layers are very different from those of bulk water. These hydration layers play a role during biological interactions between macromolecules, but it is difficult to study their atomic three-dimensional structures, as they are normally transient or affected by high thermal displacement parameters. This difficulty can be overcome by studying water clusters inside a protein. For this purpose, fatty acid binding proteins (FABPs), small proteins which act as intracellular lipid chaperones, are good models since they have a large internal cavity occupied by a fatty acid (FA) and a stable cluster of well ordered water molecules (Chmurzyńska, 2006 ▸). Unlike the biological interfaces between different proteins observed by X-ray crystallography (Rodier *et al.*, 2005 ▸), the water cluster inside FABPs has more than one very ordered hydration layer. It can therefore be used as a probe to assess the general rules governing water structures in interfaces.

**Figure 1:**
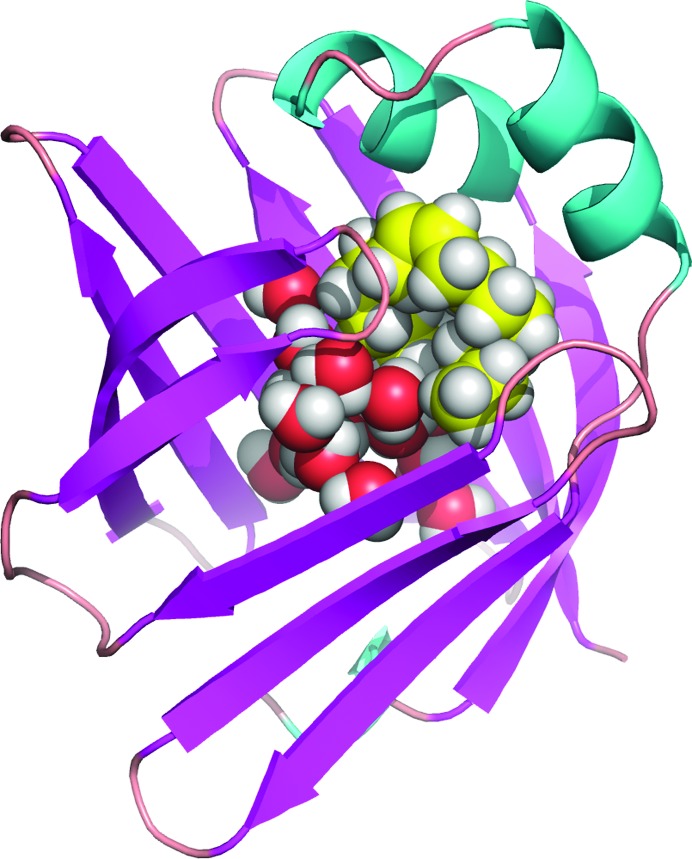
A ribbon representation of the H-FABP structure determined in this work, with β-sheets in magenta and α-helices in cyan. The internal water cluster and the oleic acid are represented as spheres occupying the internal cavity (red = O atoms, yellow = C atoms, white = H or D atoms).

FABPs coordinate lipid responses in cells and are also strongly linked to metabolic and inflammatory pathways (Haunerland & Spener, 2004 ▸; Chmurzyńska, 2006 ▸; Makowski & Hotamisligil, 2005 ▸; Coe & Bernlohr, 1998 ▸; Zimmerman & Veerkamp, 2002 ▸). FABPs are 14–15 kDa proteins that reversibly bind hydrophobic ligands, such as saturated and un­saturated long-chain FAs. All known FABPs share almost identical three-dimensional structures, including a ten-stranded antiparallel β-barrel (Chmurzyńska, 2006 ▸), which is formed by two orthogonal five-stranded β-sheets, as shown in Fig. 1 ▸. The binding pocket is located inside the β-barrel, the opening of which is framed by the N-terminal helix–loop–helix ‘cap’ domain, and FAs are bound to the interior cavity. Generally, conserved basic amino acid residues are required to bind the carboxylate head of an FA ligand in the binding pocket of an FABP (Chmurzyńska, 2006 ▸; Zimmerman & Veerkamp, 2002 ▸). The hydrocarbon tail of the ligand is lined on one side by hydrophobic amino acid residues and on the other side by ordered water molecules, which mediate the interaction between the protein and the ligand and contribute to differences in the enthalpic and entropic components of the ligand binding energy.

X-ray structures of FABPs complexed with FAs reveal that the internal cavity accommodates both the ligand and water molecules (Wiesner *et al.*, 1999 ▸; Sacchettini & Gordon, 1993 ▸). A recent study focusing on atomic resolution X-ray crystal structures of heart-FABP (H-FABP) complexed with FAs of varying alkyl chain lengths has shown that these water mol­ecules in the binding pocket can be sorted into two distinct clusters exhibiting different stabilities (Matsuoka *et al.*, 2015 ▸). The first cluster, studied in detail in the present work, is the more energetically stable and is composed of very ordered water molecules in both holo and apo H-FABP, as observed by NMR (Mesgarzadeh *et al.*, 1998 ▸) and confirmed by molecular dynamics (MD) simulations (Bakowies & Gunsteren, 2002 ▸). The second cluster is made of less stable water molecules, which are expelled by FA alkyl chains longer than 12 carbon atoms. The conserved water cluster has been reviewed (Bottoms *et al.*, 2006 ▸) and its function has been analysed (Lücke *et al.*, 2002 ▸), proposing that these water molecules form a hydration shell that interacts with the bound ligand (Scapin *et al.*, 1992 ▸; Kleywegt *et al.*, 1994 ▸; LaLonde *et al.*, 1994 ▸; Young *et al.*, 1994 ▸). In holo intestinal-FABP [I-FABP, Protein Data Bank (PDB) code 2ifb], these water molecules are located at the concave face of the slightly bent FA ligand (Sacchettini *et al.*, 1992 ▸), whereas in the holo forms of adipocyte-FABP (A-FABP, PDB code 1lie) and heart-FABP (H-FABP, PDB code 1hmr), the water molecules are clustered beneath the pseudo-re face of the U-shaped FA (LaLonde *et al.*, 1994 ▸). In these last two proteins, the surface of the binding cavity is divided into three sections, consisting of: (i) a cluster of hydrophobic side chains contacting the aliphatic chain of the ligand; (ii) a scaffold of polar and ionizable groups that interact with the bound cluster of water molecules; and (iii) a mixture of residue types near the entry portal.

The purpose of this work was to obtain a complete atomic description of the ordered water cluster and its properties in the human H-FABP–oleic acid complex and to analyse interactions between the bound ligand, the water cluster and the protein residues. To achieve this, we used a combination of high-resolution X-ray crystallography and neutron protein crystallography (NPC) to determine the atomic positions (plus alternate conformations) for the water molecules, FA atoms and protein residues, including the positions of the hydrogen atoms (as deuterium). These experiments were conducted at room temperature, thus reflecting the actual *in vivo* conditions. The resulting X-ray/neutron structure has allowed the use of the charge-density distribution, expressed in terms of multipolar components (Hansen & Coppens, 1978 ▸). These components are obtained by transfer from the ELMAMII library (Domagała *et al.*, 2012 ▸) for protein, FA and water molecule atom types. This ‘building blocks’ approach allows the accurate description of the continuous molecular electron density and the relevant derived properties of macromolecular systems, without the need for fulfilling the stringent requirements of a complete multipole refinement (Liebschner *et al.*, 2011 ▸). This transferred electron-density distribution was used to study the network of interactions formed by the protein, the ligand and the water cluster, on the basis of Bader’s quantum theory of atoms in molecules (QTAIM; Bader, 1994 ▸). As knowledge of the precise total charge distribution (nuclei positions and transferred aspherical electron density) allows the calculation of derived electrostatic properties, we performed calculations that determine the electrostatic potential being felt by the bound FA, and the electric field at the position of each internal ordered water molecule.

Note that these measurements and the corresponding calculations are not biased by the experimental methods because we study a water cluster not involved in crystallographic symmetry contacts (as it is inside a cavity) and the experiments were conducted at room temperature. Therefore, the water properties observed should be close to those *in vivo*.

## 2. Materials and methods   

### 2.1. Protein expression, purification and crystallization   

Perdeuterated H-FABP was recombinantly expressed at the Institut Laue–Langevin (ILL) Deuteration Laboratory in Grenoble, France, and purified based on the procedure described previously (Zanotti *et al.*, 1992 ▸). Briefly, *Escherichia coli* BL21(DE3) strain (Novagen), transformed with the pJexpress411 plasmid containing the synthetic cDNA coding for H-FABP, was over-expressed in perdeuterated minimal medium using *d*_8_-glycerol as carbon source (Artero *et al.*, 2005 ▸). A high cell density fed-batch culture was grown at 30°C to an OD_600_ of 8.5. H-FABP expression was then induced by the addition of 0.5 m*M* IPTG and cells (40 g wet weight) were harvested at an OD_600_ of around 11. H-FABP was purified using 25 ml of Capto Q resin (GE Healthcare). The protein was eluted with 150 m*M* NaCl, 25 m*M* Tris-HCl pH 8.0. Finally, H-FABP was purified in a Hiload 26/60 Superdex 75 gel filtration column (GE Healthcare) in 20 m*M* Tris pH 7.5, 50 m*M* NaCl. The published crystallization conditions (Young *et al.*, 1994 ▸) were optimized in terms of concentration and seeding conditions.

### 2.2. X-ray and neutron diffraction data collection of H-FABP   

A single crystal of perdeuterated H-FABP, with a radically small volume of 0.05 mm^3^ (1 × 0.25 × 0.2 mm), was mounted in a quartz capillary, surrounded on both sides by a small amount of mother liquor, and sealed with wax ready for data collection. Quasi-Laue neutron diffraction data up to 1.90 Å resolution were collected at room temperature using the LADI-III beamline (Blakeley *et al.*, 2010 ▸) at the ILL, Grenoble, France. In terms of the ratio of crystal volume (0.05 mm^3^) to asymmetric unit-cell volume (34 000 Å^3^) of the protein, this study has the smallest ratio (14 × 10^14^) of any neutron protein crystallography study thus far (Blakeley *et al.*, 2015 ▸). As is typical for a Laue experiment, the crystal was held stationary at a different φ setting for each exposure. In total, 36 images were collected (with an average exposure time of 18.6 h per image) from four different crystal orientations. The neutron data were processed using the program *LAUEGEN* modified to account for the cylindrical geometry of the detector (Campbell *et al.*, 1998 ▸). The program *LSCALE* (Arzt *et al.*, 1999 ▸) was used to determine the wavelength-normalization curve using the intensities of symmetry-equivalent reflections measured at different wavelengths. No explicit absorption corrections were applied. These data were then merged in *SCALA* (Winn *et al.*, 2011 ▸). The statistics for the neutron data collection are shown in Table S1 in the supporting information. Another perdeuterated crystal (from the same batch) was also mounted in a quartz capillary and X-ray diffraction data were collected up to 0.98 Å resolution at room temperature on the X06SA beamline at the Swiss Light Source (SLS). The statistics for the X-ray data collection are shown in Table S2 in the supporting information. The structure has been deposited with the PDB (entry 5ce4).

### 2.3. Solution and refinement of H-FABP.   

The structure was solved by molecular replacement using the model with PDB code 1hmr and refined using the *PHENIX* software suite (Adams *et al.*, 2010 ▸). The model was first refined with the X-ray terms alone, followed by joint X-ray and neutron (X+N) refinement (Afonine *et al.*, 2010 ▸). Deuterium atoms were added with the program *ReadySet* (Adams *et al.*, 2010 ▸) in an iterative process, first by modelling them on a chemical basis, followed by validation from the neutron maps. D_2_O molecules were added according to clear positive peaks in the *F*_o_ − *F*_c_ difference nuclear scattering density maps and all model modifications were made with the modelling program *COOT* (Emsley & Cowtan, 2004 ▸). A total of 222 D_2_O water molecules were added. Final X+N structural refinement statistics are shown in Table S3 of the supporting information.

### 2.4. Electron-density transfer and electrostatic computations   

Electron-density distribution of the complete X+N H-FABP crystal structure was obtained by transferring multipolar parameters stored in the ELMAMII library (Domagała *et al.*, 2012 ▸), using the *MoPro* software package (Jelsch *et al.*, 2005 ▸), to the protein and water molecules and FA atoms. The resulting electron density allows the analytical computation of derived properties such as the molecular electrostatic potential and intermolecular electrostatic interaction energies (Dominiak *et al.*, 2009 ▸; Fournier *et al.*, 2009 ▸) and can be analysed topologically in the framework of the QTAIM. Here, the topological analysis consisted of the search of (3, −1) bond critical points (BCPs) in intermolecular regions between the FA and the surrounding protein and water molecules.

In the present study, to simplify the interpretation of the electrostatic properties, only the major conformations of the two disordered water molecules (*W*51 and *W*67) present in the binding cavity were selected for the transfer procedure, with associated full occupancies. As the model was jointly refined against neutron and X-ray diffraction data, all *X*—H covalent bonds were elongated in the final model to fit the values observed in the neutron diffraction experiments, so that the *X*—H bonding electron density could be modelled by a transferred dipolar function oriented along the bond direction (Allen & Bruno, 2010 ▸). For each of the 17 studied water molecules (14 occupying the binding cavity plus three other water molecules buried in the structure but not located in the main cluster) and for the ligand, electrostatic potential maps were computed in regular three-dimensional grids using 0.05 Å sampling, without the contribution associated with the considered molecule. This way, according to the superposition principle in electrostatics, the resulting electrostatic potential is considered as that being felt by a given water molecule or by the ligand due to its environment. Electric field vectors at the positions of the water molecules were subsequently computed by numerical differentiation using the central difference method, where interpolated values are obtained by tricubic Lagrange polynomials. All charge-density related computations and representations were performed with the programs of *MoProSuite* (Jelsch *et al.*, 2005 ▸, Guillot, 2012 ▸). The electro­static interaction energies between the water molecules and their environment were computed using the exact potential and multipole methods (EP/MM) (Volkov *et al.*, 2004 ▸), as implemented in the *VMoPro* software of *MoProSuite*. The dipole moments of the water molecules were computed from the transferred charge distribution using both atomic charges (valence populations) and atomic dipole moment contributions. In the ELMAMII modelling, the permanent dipole moment of a water molecule is equal to 1.92 D. To quantify the orientation of the water molecule’s dipole moment with respect to the external electric field, we considered two angular criteria: first, the angle (α) between the electric field vector and the H—O—H plane, and second, the angle (β) between the electric field vector and the H—O—H bisecting plane. The β and α angles are thus, respectively, measures of the in-plane and out-of-plane deviations between the external electric field and the dipole moment vectors of the water molecule, the latter lying at the intersection of the considered planes. The estimation of uncertainties on charge-density derived properties is discussed in the supporting information.

## 3. Results and discussion   

### 3.1. Structure description   

Several FABP isoforms have been structurally investigated as isolated recombinant proteins by X-ray crystallography, NMR and other biochemical and biophysical techniques (Furuhashi & Hotamisligil, 2008 ▸). FABPs have an extremely wide range of sequence diversity, from 15 to 70% sequence identity between different members (Chmurzyńska, 2006 ▸). Analyses of the PDB entries 3rzy (A-FABP without FA) and 3p6c (A-FABP with citrate) (González & Fisher, 2015 ▸) show an internal water cluster which is well conserved, even when the FA molecule is not present. In this case the water in the space of the absent FA was not observed, probably due to disorder, as indicated by hydration site analysis of the *apo* form (Matsuoka *et al.*, 2015 ▸).

**Figure 2:**
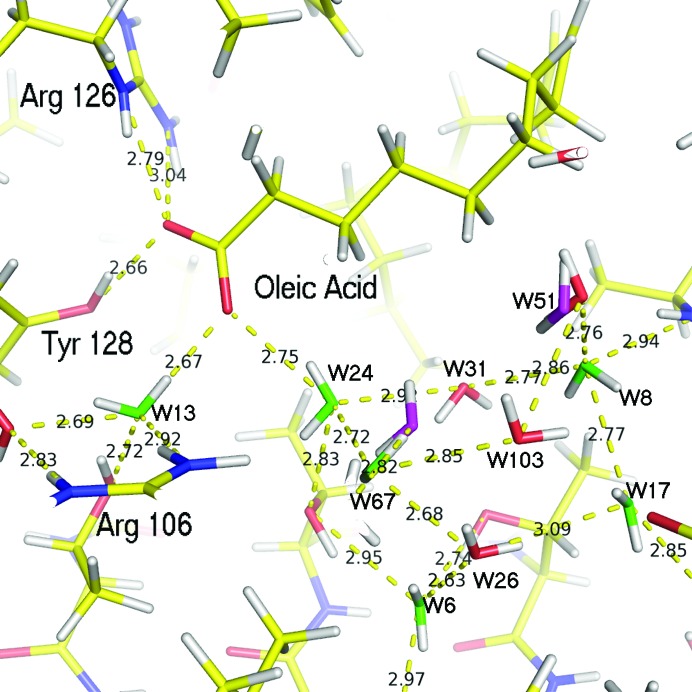
Cluster water molecules inside the cavity, with hydrogen-bond contacts indicated as yellow dashed lines (distances are given in Å). Water molecules with single occupancy and a close to tetrahedral conformation are indicated in green, and those with alternate conformations in magenta. O atoms in other water molecules are indicated in red.

In this work, the structure of perdeuterated human H-FABP was determined for the first time at room temperature with combined neutron and X-ray diffraction data to resolutions of 1.90 and 0.98 Å, respectively (for statistics of the data collection and refinement, see Tables S1–S3 in the supporting information). Analysis of the electron and nuclear scattering density maps showed that oleic acid (the FA naturally bound to H-FABP expressed in *E. coli*) does not occupy the whole internal cavity. As depicted in Fig. 1 ▸, the FA (yellow C atoms) is folded in the typical U-shaped conformation systematically observed in complexes between FABP and long-chain FAs (Smathers & Petersen, 2011 ▸; Zanotti, 1999 ▸). Its carboxylate head is in contact with the conserved Tyr128 and Arg126 side chains (Fig. 2 ▸) and, through a water bridge, with Arg106. Along with the FA, 14 water molecules fully occupy the rest of the cavity (Fig. 1 ▸), of which two are observed with double conformations. The water molecules are packed against the oleic acid and are connected with the external solvent through a narrow pore. These water molecules are very well ordered, even at room temperature (mean *B* factor for the O atoms = 15.6 Å^2^), more so than the oleic acid which presents a non-H-atom mean *B* factor of 32.5 Å^2^, ranging between 11.8 Å^2^ for the carboxyl group to 48 Å^2^ for the terminal methyl C atom.

### 3.2. Water cluster analysis   

#### 3.2.1. Hydrogen-bonding network   

**Figure 3:**
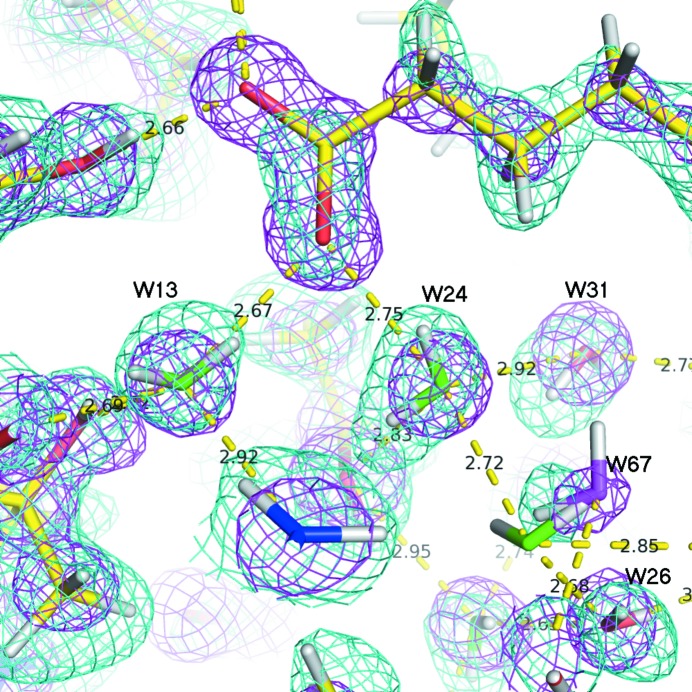
Cluster water molecules inside the cavity, with electron and nuclear scattering density maps. Cyan: 2*F*_o_ − *F*_c_ neutron map contoured at 1.7 r.m.s.; magenta: 2*F*_o_ − *F*_c_ electron density map contoured at 2.0 r.m.s.. Tetrahedral water molecules with single occupancy and a close to tetrahedral conformation are indicated in green. O atoms in other water molecules are indicated in red. Dashed lines indicate hydrogen bonds (distances are given in Å)

Fig. 3 ▸ shows the electron (X-ray) and nuclear (neutron) scattering density maps for the internal water molecules, revealing the positions of their H atoms (as deuterium), and thus their orientation. We first use standard geometric criteria to locate hydrogen bonds involv­ing water molecules in the ordered cluster. They are linked in a network showing mostly tetrahedral coordination (Fig. 2 ▸). The geometries of the hydrogen bonds in this network are described in detail in Table S4 in the supporting information. The hydrogen-bond distances between the oxygen and acceptor atoms of the ordered water molecules show a wide range of values between 2.68 and 3.05 Å, with donor group–acceptor angles systematically greater than 100°. However, these hydrogen bonds are on average rather short, with a mean O⋯O distance of 2.83 Å, leading to a mean volume of 17.2 Å^3^ per water molecule inside the cavity (the cavity volume was calculated with the program *McVol*; Till & Ullmann, 2010 ▸). This can be compared with a van der Waals water molecule volume between 16 and 18 Å^3^ and an average volume of 30 Å^3^ for bulk water at 24°C. Therefore, the water molecules are quite tightly packed in the binding cavity of H-FABP. All 14 of the water molecules in the cluster are involved in at least two hydrogen bonds as donors and two others as acceptors, corresponding to tetrahedral coordination with at least one chemical group from the protein. An additional three buried water molecules do not belong to the cluster. Among them, *W*1 is a resident water molecule conserved among nine different members of the FABP protein family and is presumed to play a structural role in the stabilization of the folded protein (Likić *et al.*, 2000 ▸). It has a nearly flat coordination, being involved in one hydrogen bond as acceptor with the amide H atom of the Val84 main chain and in two hydrogen bonds as donor with the main chain O atoms of Val68 and Lys65. Two cluster water molecules (*W*13 and *W*24) are in contact with atom O2 of the FA carboxylate head, forming strong hydrogen bonds with O⋯O distances of 2.68 and 2.76 Å, respectively. These are among the shortest hydrogen bonds involving water molecules, which is consistent with the fact that they are known to play a role in FA binding; they are indeed systematically found at quasi-identical positions in H-FABP and muscle-FABP (M-FABP) structures complexed with FAs [see PDB codes 3wvm, 4tkj, 4tkh, 4tkb and 4tjz (Matsuoka *et al.*, 2015 ▸); 1hmr, 1hms and 1hmt (Young *et al.*, 1994 ▸)], and also at slightly displaced positions in other members of the FABP family (such as 4bvm; Ruskamo *et al.*, 2014 ▸).

Only three other cluster water molecules (*W*51, *W*7 and *W*31) interact, through weak C—H⋯O hydrogen bonds, with the H atoms bound to atoms C3, C5, C6 and C18 of the FA. *W*31, located at the top of the U-shape of the FA, acts as a C—H⋯O hydrogen-bond acceptor in three interactions, with H⋯O distances of 3.46 (donor group is C5—H52), 3.38 (C3—H31) and 2.55 Å (C18—H181). This way, the conserved *W*31 water molecule bridges both extremities of the FA, clearly contributing to stabilizing its folded ‘U’ conformation. Another C—H⋯O interaction links non-cluster *W*28 (located on the other side of the U formed by the ligand) and the C14—H141 hydrogen atom of the FA. To summarize, it appears that, from a hydrogen-bond geometry perspective, the FA alkyl chain forms few interactions with the water cluster. This observation agrees with the recent finding by Matsuoka and co-workers, who have shown in a convincing way that the energetic stability of the water molecules in this cluster prevents long FAs from folding correctly in the binding pocket (Matsuoka *et al.*, 2015 ▸). Hence, this cluster presents an intrinsic stability independent of the formation of strong interactions with the aliphatic chain of the FA.

#### 3.2.2. Alignment between electric field and water mol­ecule dipoles   

**Figure 4:**
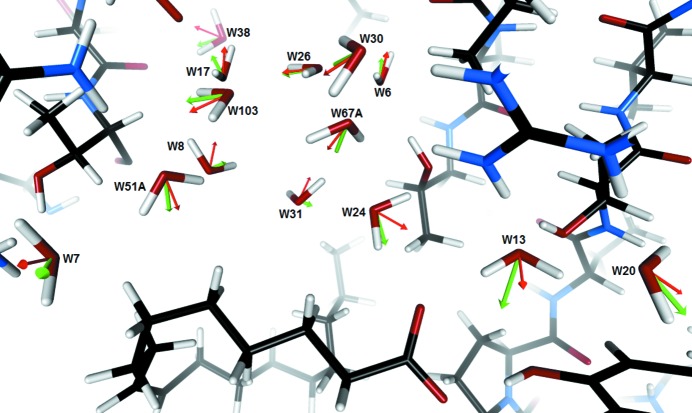
Partial view of the water cluster filling the binding pocket along with the FA. Water molecule dipole moments are represented as thin red arrows, with the scale 1 Å = 2 debye. Electric field vectors computed at the water molecules’ centres of mass are represented as green arrows, using the scale 1 Å = 0.1 e Å^−2^ = 14.4 GV m^−1^. The oleic acid ligand can be seen at the bottom of the picture.

As expected, the water molecules buried in this cavity are exposed to strong electric fields, ranging between 6.6 (7) and 21.4 (8) GV m^−1^ when computed using the vacuum dielectric constant. We studied the relation between the electric field and the orientations of the water molecule dipole moments, defined in terms of the two angles α and β (§2.4). For the 17 water molecules included in the analysis (14 in the cluster and three buried ones), it appears that both measured angles are significantly smaller than 90°, with average values of 20° and 23° for α and β, respectively (Fig. 4 ▸, Table 1 ▸). There are no examples, even taking into account the estimated uncertainties on these angles, where both vectors show inverted directions (angles larger than 90°), meaning there is a clear correlation between them. Water molecules hydrogen-bonded in a tetrahedral coordination are expected to be located in a close dipolar environment, where positive charges correspond to H atoms interacting with the water O atom, and negative charges to electronegative atoms accepting hydrogen bonds with the water protons. However, surprisingly, the most favourable cases, *i.e.* where both angles are close to zero, do not necessarily correspond to ideal tetrahedral coordination of the water molecules. For example, this is the case for a water molecule (*W*28) that is in contact, on its oxygen side, with the oleic acid molecule and located in the narrow pore connecting the binding pocket with the external solvent molecules. This water molecule is involved (as an acceptor) in only one clear hydrogen bond (with the Arg126 side chain) but nevertheless presents a nearly perfect alignment with the external electric field (Table 1 ▸). This implies that the alignment is not driven by the local environment alone but by the overall electric field.

Furthermore, water molecules that are subjected to a stronger external electric field present a better alignment between their dipole and the field vector computed at their centre of mass (Table 1 ▸). Conversely, a weaker electric field corresponds to a larger observed angle. This trend can be observed for all the studied water molecules except for *W*13: excluding *W*13, the correlation coefficient between the electric field/dipole moment raw angle and the corresponding electric field values reaches 0.76, but it drops to 0.56 if *W*13 is included in the statistics (Fig. S2 in the supporting information). However, this can be explained by the peculiarities of the *W*13 environment. This conserved water molecule is tightly packed between the negatively charged carboxylate head of the FA and the basic side chain of Arg106, and is hence located in a region of strong external electric field. Moreover, it makes the shortest donor hydrogen-bond interactions of all considered water molecules (with the O atom of *W*20, O⋯O = 2.69 Å, and with atom O2 of the fatty acid, O⋯O = 2.68 Å) and among the shortest as hydrogen-bond acceptor (with the Arg106 and Thr40 side chains; Table S4 in the supporting information). This may indicate that the formation of strong hydrogen bonds, especially as hydrogen-bond donor, can overcome the torque effect of a misalignment with the external electric field. The opposite phenomenon can be illustrated by the case of water molecule *W*3, which is also subjected to a strong electric field but forms comparatively weaker donor hydrogen bonds (with the main-chain O atoms of Leu104 and Leu91, O⋯O = 2.99 and 2.85 Å, respectively). Consequently, *W*3 shows a good alignment of its dipole moment with the electric field.

It has been already shown by molecular dynamics simulations that the average reorientation time of water molecules located within 7 Å of the protein surface is significantly longer than that of bulk water (Rocchi *et al.*, 1998 ▸). Hence, even if the orientation of water molecules depends on many factors, such as steric constraints or hydrogen bonding, the reorientation time may be increased by the restriction of the rotational freedom of interfacial water molecules by the dipole/field alignment effect we characterize in this study. This result can be linked to the decrease in the relative dielectric constant of such water clusters when compared with bulk water. The relative weights of the hydrogen-bonding and dipole-alignment effects might vary according to each case, as shown by the comparison between *W*3 and *W*13, and it seems that, when a water molecule is not constrained by the formation of strong hydrogen bonds like *W*13, its tendency to align according to the felt electric field appears stronger.

#### 3.2.3. Electrostatic interaction energies   

The intrinsic stability of the embedded water cluster was studied by evaluating the electrostatic interaction energies with their environment. In order to characterize the relative contribution of the ligand charge distribution to these energies, the computations were performed in two stages, with and without its contribution. A comparison of the electrostatic interaction energies (Table 2 ▸) in both situations confirms the stability of the water cluster. As expected, the two water molecules *W*13 and *W*24, which are strongly hydrogen-bonded and in close contact with the negatively charged carboxylate head of the FA, undergo a significant destabilization in the absence of the ligand (Δ*E*_elec_ = 11 and 16 kcal mol^−1^, respectively; 1 kcal mol^−1^ = 4.184 kJ mol^−1^). All the other water molecules in the cluster show either a weak destabilization (largest Δ*E*_elec_ = 2 kcal mol^−1^ for *W*51) or a weak stabilization (largest Δ*E*_elec_ = −2 kcal mol^−1^ for *W*20). Contrary to the cases of *W*13 and *W*24, whose destabilization appears to be clearly significant, the electrostatic interaction energies for the 12 other water molecules in the cluster vary by amounts that are lower than, or of the same order of magnitude as, the estimated errors on these quantities. These results agree with the cluster observed in the atomic resolution apo form of an adipocyte FABP4 (PDB code 3rzy; González & Fisher, 2015 ▸), which shows water molecules at similar positions to *W*3, *W*6, *W*7, *W*8, *W*17, *W*31, *W*26 and *W*51 (Fig. S3 in the supporting information) observed in the present study. Hence, it appears that, apart from *W*13 and *W*24, the water cluster is inherently stable, and from an electrostatic interaction energy perspective the presence of the FA does not significantly influence its stability. Again, this confirms that this water cluster is an inherent part of the H-FABP structure, and apart from the formation of hydrogen bonds with the polar head of the ligand, its role may be limited to an exclusion factor for ligands whose alkyl chain is too long (Matsuoka *et al.*, 2015 ▸).

### 3.3. Ligand binding   

#### 3.3.1. Electrostatic environment of the fatty acid   

**Figure 5:**
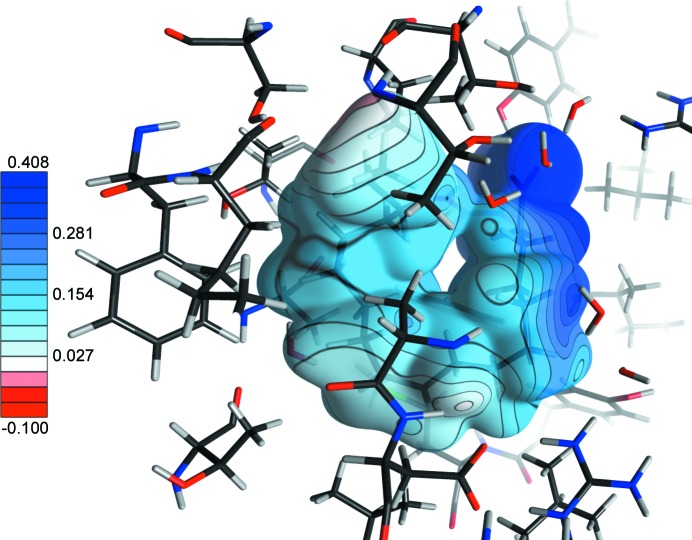
0.01 e Å^−3^ total electron-density isosurface of the FA in the binding pocket, mapped by the electrostatic potential (e/Å) generated by the whole protein, including explicit water molecules.

The electrostatic environment of the bound FA was also analysed. As expected for a negatively charged ligand, the electrostatic potential generated by the whole hydrated protein at the surface of the ligand is globally electropositive (Fig. 5 ▸). A clear electrostatic complementarity is observed, where the negatively charged FA carboxylate group interacts with the electro­positive potential of the basic Arg126 and Arg106 side chains. Ruskamo and co-workers reported that, for their 0.93 Å resolution X-ray structure of A-FABP in complex with palmitate, Arg106 appeared unprotonated on one amine group, leading to a neutral side chain (Ruskamo *et al.*, 2014 ▸). We do not observe the same phenomenon here: both arginine side chains are clearly protonated and contribute to a strong electropositive potential. As mentioned above, one side of the hydrocarbon U-shaped tail of the FA is in contact with the side chains of hydrophobic residues, where the electrostatic complementarity is less obvious, as the slightly positive charges of side-chain H atoms are in contact with similarly charged H atoms of the FA. These residues contribute to a weaker but still electropositive potential, nevertheless accommodating the low positive charges of the FA hydrocarbon H atoms. The sole exception occurs for the slightly electronegative environment of the ligand terminal methyl group. This is due to the nearby proximity of the main-chain carbonyl O atoms of Thr53 and Lys58, which are involved in C—H⋯O hydrogen bonds with the FA methyl group. In the ELMAMII modelling, methyl H atoms are slightly more positively charged than H atoms of CH_2_ types (partial charges are 0.041 and 0.037 e, respectively), resulting in a weak positive-charge accumulation at the terminal methyl group of the alkyl chain.

To summarize, we observe then a very fine electrostatic complementarity between the charge distribution characterizing long-chain FAs and various regions of the binding pocket. The complementarity observed in the methyl part of the FA may be linked to the better affinity of FABPs for ligands which are long enough to allow the ‘U-shaped’ conformation, bringing the terminal methyl to an electrostatically favourable region.

#### 3.3.2. Topological analysis   

Intra- and intermolecular interactions can be precisely characterized and quantified by performing a topological analysis of electron-density distribution in the framework of the QTAIM approach, developed by Bader (1994 ▸). Studying bonding interactions in this approach consists of analysing the topology of the total electron density by searching ridges, termed bond paths, of maximal value between nuclei, mirroring lines of maximally negative potential energy density (Bader, 1998 ▸). On such an interatomic (actually internuclei) bond path lies a point of special importance, named the bond critical point (BCP), where the electron density displays a saddle-type curvature, *i.e.* is minimal along the bond path. It has been shown by Bader and coworkers, and exploited in numerous studies (Matta, 2007 ▸), that the existence of a bond path bridging atoms, and an associated BCP, is a ‘*universal indicator of chemical bonding of all kinds: weak, strong, closed-shell, and open-shell interactions*’ (Matta, 2007 ▸). Indeed, values of the electron density ρ(*r*_cp_) and of its Laplacian ∇^2^ρ(*r*_cp_) (*i.e.* the sum of its second derivatives) on the BCP allow one to determine the type of interaction and quantify its strength. For instance, covalent bonds are characterized by a negative value of the Laplacian, while in closed-shell bonding (*e.g.* hydrogen bonds) the depletion of the electron density in the interatomic region leads to a positive Laplacian. The strengths of various types of closed-shell interactions, measured in terms of electron-density properties at the BCP, have been extensively studied by Mata *et al.* (2010 ▸). In particular, they showed that their dissociation energies *D*_e_ can be estimated from the value of the electronic potential energy density *V*(*r*_cp_) at the BCP (Espinosa & Molins, 2000 ▸), which is accessible from the values of ρ(*r*_cp_) and ∇^2^ρ(*r*_cp_) using the Abramov formula (Abramov, 1997 ▸).

In this study, the knowledge of an accurate total charge distribution, made up of precise nuclei positions (including H atoms) and the transferred aspherical electron density, definitely makes the QTAIM approach the method of choice to analyse, at an atomic level, intermolecular interactions in the H-FABP complex. Hence, a topological analysis of the transferred electron density was performed to search for interatomic interactions between atoms of the bound ligand and its environment, *i.e.* of the protein and water molecules. All interactions found by locating a saddle BCP and an associated bond path are summarized in Table 3 ▸. We can distinguish four main categories of interatomic contacts involving the FA: (i) hydrogen bonds with carboxylate O atoms as acceptors; (ii) C—H⋯O hydrogen bonds between FA H atoms and water O atoms; (iii) a C—H⋯π hydrogen bond involving the π electrons of the oleic acid C=C double bond (oleic acid presents a single unsaturation at the ω9 position); and (iv) 35 contacts between H atoms, including two intramolecular ones. All hydrogen bonds shown by geometric criteria were confirmed by the localization of a bond path and a saddle critical point.

**Figure 6:**
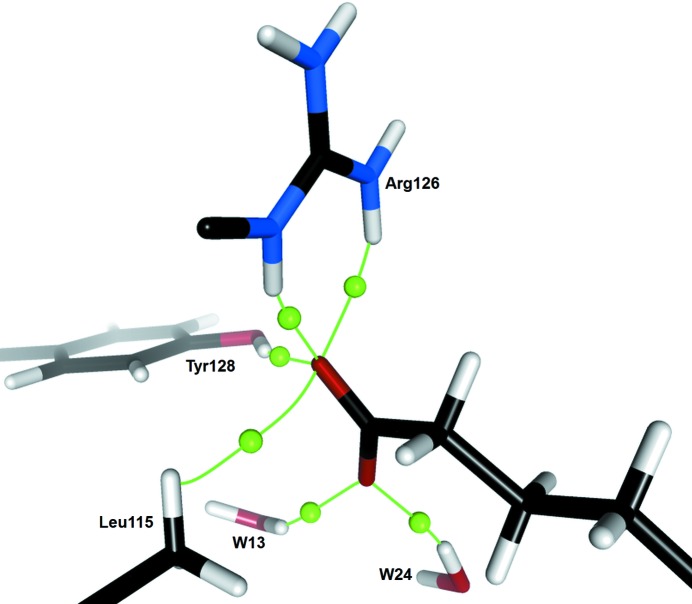
Bond critical points and associated bond paths (pictured in green) of hydrogen bonds involving the O atoms of the FA carboxylate head.

The carboxylate head of the FA accepts a total of six hydrogen bonds, whose bond paths and critical points are depicted in Fig. 6 ▸. Carboxylate atom O1 acts as acceptor in highly bifurcated hydrogen bonds, combining the very strong O—H⋯O1 bond with the Tyr128 hydroxyl group, two N—H⋯O1 bonds with the guanidinium group of Arg126, and a weak C—H⋯O1 interaction with an H atom of the Leu115 side chain. On the other side, atom O2 interacts only with *W*24 and *W*13 through O—H⋯O2 hydrogen bonds. Three of these interactions present H⋯O (1, 2) distances between 1.73 and 1.9 Å, reflecting the strength of the FA carboxylate-group binding in the FABP cavity. Using the relationship between the dissociation energy (*D*_e_) of a hydrogen bond and the electron density and Laplacian values at the corresponding BCP (Espinosa *et al.*, 1998 ▸; Mata *et al.*, 2010 ▸), the total *D*_e_ of the interactions involving the FA polar head reaches 35 kcal mol^−1^. The enthalpy gain upon oleic acid binding by H-FABP, measured by calorimetric methods (Matsuoka *et al.*, 2015 ▸), reaches Δ*H* = −20.9 kcal mol^−1^; even if this value cannot be directly compared with the estimated total *D*_e_, their relative magnitudes indicate clearly the preeminent contribution to the protein–ligand binding affinity of these few interactions involving the polar head of the FA.

**Figure 7:**
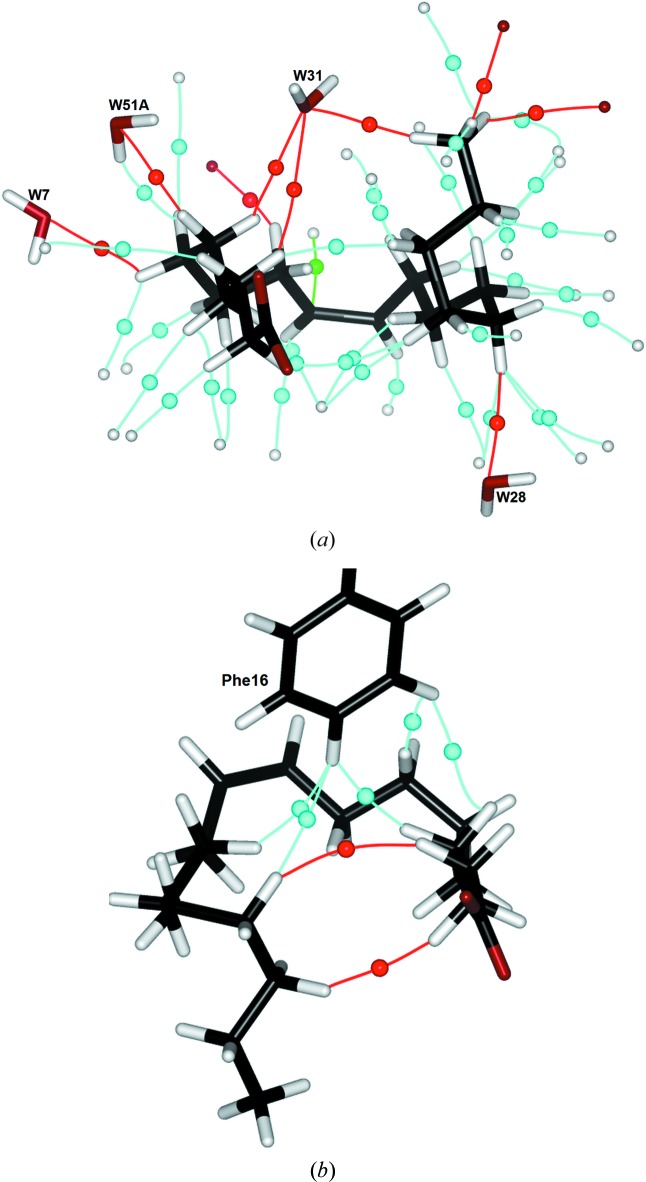
(*a*) Bond critical points and associated bond paths of H⋯H (light blue), C—H⋯O (red) and C—H⋯π (green) hydrogen bonds. For the sake of clarity, only protein atoms involved in the interactions are represented (as grey spheres for H atoms and red spheres for O atoms). Hydrogen bonds involving the carboxylate group of the FA are represented in Fig. 6 ▸ and thus omitted from this picture. (*b*) Bond critical points and associated bond paths of H⋯H bonds between the FA and Phe16 side chain (light blue), and FA intramolecular H⋯H bonds (red).

For such a molecule containing numerous CH groups, the formation of the C—H⋯O hydrogen bonds commonly encountered in proteins is favoured, especially with water O atoms, and therefore could be expected to be a significant contributor to the stabilization of the FA alkyl chain (Sarkhel & Desiraju, 2004 ▸). However, this is not the case here, as the ligand alkyl chain is mostly in contact with the H atoms of the hydrophobic residues pointing into the binding pocket. This is the case for all FA C atoms except C1–C6, which line the cavity occupied by the water cluster, and methyl atom C18, which ends up near *W*31 due to the U-shaped fold of the alkyl chain bringing the FA tail close to its head. As a consequence, the FA tail forms only nine interatomic contacts of C—H⋯O type located by the mean of a bond path and a saddle BCP (Table 3 ▸). Among these nine contacts, three involving the O atoms of *W*31 and *W*28 present long H⋯O distances (H⋯O > 3.3 Å) and consequently low electron-density values at the corresponding BCP [ρ(*r*_cp_) < 0.008 e Å^−3^]. However, it must be noted that these weak contacts were located, with similar electron-density values at the BCP, in all the perturbed models accounting for uncertainties on atomic coordinates and charge-density parameters generated to estimate the standard error on the electron-density derived properties (see §2.4). For this reason, even if they can hardly be defined as true C—H⋯O hydrogen bonds, they can nevertheless be considered as actual water–ligand weak stabilizing interactions. Using the potential energy density, which can be estimated from ρ(*r*_cp_) and ∇^2^ρ(*r*_cp_) at their BCP, each of these weak contacts presents a bond energy of ∼1 kcal mol^−1^, *i.e.* about 30% of that of a standard C—H⋯O hydrogen bond. The other six C—H⋯O interactions display H⋯O distances between 2.55 and 2.86 Å, so they satisfy the distance criteria defining C—H⋯O hydrogen bonds (Sarkhel & Desiraju, 2004 ▸). Three of these interactions involve the H atoms of the FA methyl group, interacting with, respectively, the Lys58 main chain, the Asp76 side chain and the *W*31 O atom. Six of the nine C—H⋯O hydrogen bonds involve water H atoms, and for this category of interaction the role of *W*31 appears noteworthy. This sole water molecule is in fact responsible for three of the nine C—H⋯O contacts and half of those involving water O atoms. *W*31 is indeed located, and properly oriented, at a position allowing it to interact with both ends of the FA alkyl chain: two weak C—H⋯O contacts with FA atoms H31 and H52 bound to, respectively, atoms C3 and C5 (*i.e.* located near the polar head), and a C—H⋯O hydrogen bond with atom H181 of the FA methyl terminal group. Hence, it appears that *W*31 is ideally positioned to stabilize the U-shaped conformation of the FA, by bridging the tail of the molecule to a position located near its head, as seen in Fig. 7 ▸(*a*).

The most striking feature of the topological analysis of interactions between the FA and the protein is the presence of 35 C—H^δ+^⋯^δ+^H—C intermolecular interactions between the H atoms of the oleic acid molecule and of the hydrophobic side chains, as well as two intramolecular ones linking pairs of H atoms located at both ends of the alkyl chain (Table 3 ▸). These interactions form an intricate network, visible through their curved associated bond paths, represented along their corresponding critical points in Fig. 7 ▸(*a*). These 35 H⋯H contacts involve 27 of the 33 H atoms of the FA, meaning that some of them present a bifurcated geometry (H9, H10, H41, H111, H171 and, again, H183, which is also involved in contacts with the Lys58 main-chain O atom), and a trifurcated one for H141. However, they are distributed evenly along the alkyl chain, from H22 on atom C2 to those of the C18 terminal methyl group. Obviously, the presence of these contacts is a direct consequence of the packing of the FA on the hydrophobic side of the binding pocket. As expected, a large majority (27) of the 35 H⋯H contacts shown by the presence of a bond path display internuclei distances larger than the sum of the H atoms’ van der Waals radii (*r*_H_ ≃ 1.2 Å; Bondi, 1964 ▸). These interactions are characterized by ρ(*r*_cp_) values between 0.002 and 0.03 e Å^−3^, while the ∇^2^ρ(*r*_cp_) values lie in the range 0.04–0.4 e Å^−5^. Again, the low range in electron density value appears very small, but these interactions were found topologically in each of the models used to represent the degrees of uncertainty of the atomic coordinates. Such contacts, whose internuclei distance is larger than twice the H-atom van der Waals radius, can be classified as weak stabilizing van der Waals interactions (Wolstenholme & Cameron, 2006 ▸). Even if, individually, each of the weak H⋯H interactions contributes only moderately, they may collectively have a significant impact on the H-FABP–FA binding energy. This is in agreement with the observation by Matsuoka *et al.* (2015 ▸) that the enthalpic gain upon FA binding by H-FABP tends to increase with the size of the alkyl chain, up to a chain-length limit imposed by the stable water cluster. The shortest H⋯H contact (H⋯H = 2.57 Å) falling within this category is intramolecular, between atoms H31 and H161 located near, respectively, the head and the tail of the FA. Hence, similar to the C—H⋯O interactions with the *W*31 O atom, this H⋯H interaction could contribute to stabilizing the closed conformation of the fatty acid. The Phe16 residue forms the largest number of such ‘long’ H⋯H bonds with the FA tail (Fig. 7 ▸ *b*). Its side chain points to the top of the pseudo-si face of the FA, perpendicular to the plane formed by atoms C1–C16 defining the U conformation. The FA wraps around a line going through the Phe16 C*G* and C*Z* atoms, locating its H*Z* and H*E*1 atoms at less than 3 Å from the H atoms on atoms C4, C7, C12 and C15 of the FA. This structural arrangement allowed the suggestion by Zanotti and co-workers that Phe16 ‘*may be a key determinant in FA specificity and affinity in M-FABP*’ (Zanotti *et al.*, 1992 ▸). This was later confirmed by directed mutagenesis experiments, where Phe16 was mutated into tyrosine, serine (which are less prone to forming H⋯H bonds due to the presence of the polar hydroxyl H atom) or valine (which is significantly less bulky than phenylalanine) residues, resulting in all cases in a significant drop in the oleic acid binding activity (Volkov *et al.*, 2004 ▸). In the present study, the observed H⋯H bonds are favoured by the orientation of the Phe16 side chain with respect to the U-shaped FA, as shown by the BCPs and bond paths represented in Fig. 7 ▸(*b*). Again, the position of the Phe16 side chain favouring the formation of several H⋯H bonds with both sides of the FA alkyl chain may be an explanation, at a detailed atomic level, of the important role of Phe16 in FA binding in the FABP binding pocket.

Among these H⋯H interactions listed in Table 3 ▸, ten are especially noteworthy as they present internuclear distances lower than the sum of the van der Waals radii of the interacting H atoms, so that they could be considered as ‘steric non-bonded repulsive’, while being counterbalanced by the other stabilizing H⋯H contacts which appear, in this structure, to be more numerous. However, such H⋯H interactions (not to be confused with dihydrogen bonding or hydride bonds) have already been studied by means of the AIM theory. Matta and co-workers showed that these interactions, where two H atoms bearing the same or similar weak positive charges (typically C—H hydrogen atoms) come in close proximity to allow the formation of a bond path, lead to a local stabilizing contribution to the molecular energy (Matta *et al.*, 2003 ▸). This stabilizing contribution has also been shown by other studies. Wolstenholme & Cameron (2006 ▸) compared the topological properties of H⋯H bonds with those of conventional hydrogen bonds, and classified them as weak favourable interactions (Koch & Popelier, 1995 ▸). The relationship existing between the number of H⋯H bonds formed between branched alkanes and their corresponding boiling points has been shown (Monteiro & Firme, 2014 ▸). In the present study, H⋯H bonds found by topological analysis of the total electron density fall within the stabilizing interactions shown by Matta *et al.*, with internuclear distances less than 2.4 Å, and electron density ρ(*r*_cp_) and Laplacian ∇^2^ρ(*r*_cp_) at the BCPs greater than 0.03 e Å^−3^ and 0.3 e Å^−5^, respectively. From this point of view, one could consider that these short H⋯H bonds contribute to stabilizing the FA conformation, and consequently also to favouring its binding with H-FABP.

## 4. Conclusions   

In this study we have used both X-ray and neutron diffraction data to determine the structure of an H-FABP–oleic acid complex at room temperature. The use of a tiny perdeuterated crystal (0.05 mm^3^) allowed us to locate the deuterium atoms of the ordered water molecule cluster bound inside an internal pocket, together with the FA. On the basis of this structure, we have then performed electrostatic calculations and electron-density topological analysis using a transferred aspherical charge distribution to analyse the internal water cluster and the interactions between the bound FA, the water molecules and the protein atoms.

From this analysis, we can extract three main conclusions:

(i) The internal cluster of 14 water molecules presents an inherent stability and seems to contribute moderately to the stabilization of the FA binding by the formation of a few weak C—H⋯O interactions. This agrees with recent results (Matsuoka *et al.*, 2015 ▸) suggesting that the role of this cluster is to discriminate between correctly sized and too long FAs, or too rigid ligands, rather than a stabilizing one. However, the structurally conserved water molecule *W*31 is ideally positioned to interact with both ends of the FA, presumably contributing to stabilizing its U-shaped conformation.

(ii) On the basis of the transferred charge distribution, we observed a striking electrostatic complementarity between the binding pocket and the bound FA, especially for the carboxyl­ate head and the terminal methyl group. The aliphatic tail of the FA is mostly in contact with hydrophobic residues, allowing the formation of numerous intermolecular H⋯H bonds as well as two intramolecular ones, revealed by the presence of BCPs and bond paths. Most of these H⋯H bonds can be classified as weak van der Waals interactions and together they contribute collectively to the stabilization of the observed FA conformation.

(iii) Within the cluster, the positions and orientations of the water molecules are strongly determined by the alignment of the water dipoles along the electrostatic field of the hydrated protein.

The hydration layers around proteins fulfil multiple roles and can have several states, which are difficult to study in three dimensions because of the inherent disorder in the transition to bulk water. By focusing on the internal water cluster of H-FABP, we have been able to observe in high detail the alignment of the water dipoles with the surrounding electrostatic field. This point might possibly be extrapolated to the ensemble of the hydration layers, explaining the observation that the mobility of water molecules in these layers is strongly restricted and therefore significantly different from bulk water, in which there is no defined orientation (the mean dipole moment is zero). The alignment of the water dipoles along the electrostatic field could give particular properties to protein hydration layers, extending and eventually modulating the electrostatic properties of the protein surface. Note that this should in particular be the case during the formation of protein complexes, since hydration water molecules become confined in the interface between the protein surfaces, and therefore should have properties similar to those observed in the internal cavity of the FABPs. Such strong alignment implies a much lower dielectric constant, and gives a structural basis to the longer-range electrostatic interactions necessary for the formation of protein complexes.

## Supplementary Material

PDB reference: RT X+N structure of H-FABP, 5ce4

Supporting information file. DOI: 10.1107/S2052252515024161/tj5008sup1.pdf

## Figures and Tables

**Table 1:** Electric field magnitudes, and angles between the electric field and the water molecule dipole moments, measured at the water molecules’ centres of mass See §2.4 for the definition of the angles α and β and for the estimation of uncertainty values. The raw angle is that between the water molecule dipole moment and the electric field vector.

Water molecule label	Electric field magnitude (GV m^−1^)	Raw angle (°)	α angle (°)	β angle (°)	Water O-atom *B* factor (Å^2^)
1	16.6 (7)	31 (4)	26 (4)	15 (3)	10.6
3	19.6 (6)	9 (2)	8 (4)	3 (2)	12.4
6	11.3 (6)	50 (5)	17 (6)	45 (5)	11.5
7	11.5 (7)	27 (4)	7 (7)	26 (4)	11.7
8	8.9 (7)	45 (5)	13 (6)	42 (5)	12.8
13	21.4 (8)	62 (3)	58 (3)	14 (4)	11.6
17	11.1 (7)	58 (5)	40 (5)	34 (4)	14.3
20	18.6 (9)	15 (3)	5 (5)	14 (3)	17.8
24	16.3 (7)	51 (5)	39 (5)	27 (3)	14.5
26	13.7 (7)	22 (6)	5 (8)	21 (6)	22.8
28	18.3 (7)	11 (5)	9 (7)	6 (4)	24.2
30	18.2 (5)	31 (5)	12 (6)	29 (4)	20.4
31	6.6 (7)	78 (7)	60 (6)	27 (5)	13.4
38	16.2 (7)	41 (4)	32 (5)	23 (4)	15.5
51	16.0 (8)	17 (4)	15 (7)	8 (4)	19.4
67	18 (1)	19 (5)	2 (5)	19 (5)	29.7
103	17.4 (7)	35 (3)	0 (5)	35 (3)	28.2

**Table 2:** Electrostatic interaction energies (kcal mol^−1^) of the 14 water molecules in the cluster with their environment, computed with (left column) and without (right column) the FA charge-density contribution, along with their estimated uncertainties in parentheses

Water molecule label	*E*_elec_ with FA contribution	*E*_elec_ without FA contribution
3	−29 (1)	−30 (1)
6	−25 (3)	−26 (2)
7	−26 (1)	−27 (1)
8	−17 (1)	−18 (1)
13	−33 (2)	−22 (2)
17	−19 (2)	−19 (2)
20	−29 (2)	−31 (2)
24	−23 (2)	−7 (2)
26	−20 (2)	−21 (2)
30	−35 (1)	−34 (2)
31	−7 (1)	−8 (1)
51	−29 (2)	−27 (2)
67	−17 (2)	−16 (1)
103	−24 (3)	−24 (3)

**Table 3:** Summary of interactions involving the FA and their topological properties: distances between interacting atoms (Å), and values of electron density (in e Å^−3^) and Laplacian (in e Å^−5^) at the corresponding bond critical point Values in parentheses are standard errors obtained as described in the supplementary information.

Residue atom		FA atom	Distance (Å)	ρ(*r*_cp_)	∇^2^ρ(*r*_cp_)
Intermolecular H⋯H contacts					
H*D*2	Phe57	H131	1.91	0.08 (1)	1.0 (1)
H*G*22	Thr53	H182	2.12	0.055 (3)	0.87 (2)
H*B*3	Ala75	H112	2.21	0.049 (3)	0.58 (3)
H*G*21	Val25	H9	2.22	0.041 (3)	0.48 (4)
H*G*3	Pro38	H152	2.23	0.049 (3)	0.56 (4)
H*Z*	Phe16	H42	2.29	0.033 (4)	0.37 (4)
H*D*11	Leu117	H22	2.34	0.036 (2)	0.36 (2)
H*E*2	Tyr19	H62	2.38	0.030 (2)	0.34 (3)
H*B*1	Ala33	H122	2.43	0.032 (3)	0.39 (3)
H*B*2	Lys58	H172	2.49	0.026 (2)	0.27 (3)
H*D*12	Leu23	H72	2.59	0.022 (3)	0.29 (4)
H*B*2	Lys58	H183	2.61	0.021 (3)	0.30 (3)
H*Z*	Phe16	H121	2.65	0.017 (2)	0.18 (2)
H*G*2	Met20	H9	2.69	0.017 (1)	0.23 (1)
H*G*21	Thr53	H162	2.72	0.014 (1)	0.19 (1)
H*G*21	Thr36	H142	2.74	0.015 (1)	0.15 (1)
H*E*1	Phe16	H41	2.75	0.0140 (9)	0.23 (2)
H*G*11	Val25	H10	2.76	0.014 (1)	0.13 (2)
H*Z*	Phe16	H151	2.79	0.0141 (9)	0.16 (1)
H*B*1	Ala33	H141	2.82	0.011 (1)	0.16 (2)
H*G*22	Thr29	H10	2.82	0.0116 (4)	0.127 (8)
H*E*2	Phe57	H111	2.83	0.012 (1)	0.123 (7)
H*G*23	Thr60	H183	2.89	0.011 (1)	0.15 (2)
H*A*	Ala33	H141	2.91	0.009 (1)	0.13 (1)
H*E*1	Phe16	H71	2.91	0.0107 (7)	0.139 (1)
H*D*13	Leu117	H41	2.91	0.012 (1)	0.133 (8)
H*B*3	Ser55	H171	2.93	0.0096 (6)	0.105 (5)
H*B*3	Pro38	H171	2.95	0.0100 (6)	0.112 (4)
H*B*1	Ala75	H132	3.18	0.0054 (5)	0.079 (4)
H*B*	Thr74	H61	3.24	0.0077 (3)	0.064 (2)
H*B*	Thr36	H141	3.30	0.0046 (2)	0.057 (2)
H*G*3	Lys58	H111	3.34	0.0050 (4)	0.048 (3)
H*D*23	Leu104	H32	3.56	0.0026 (1)	0.040 (2)
Intramolecular H⋯H contacts					
H162	Ola133	H31	2.57	0.021 (2)	0.27 (2)
H21	Ola133	H151	3.08	0.007 (1)	0.082 (6)
C—H⋯π hydrogen bond					
H*B*3	Asp76	C9	2.79	0.039 (2)	0.38 (2)
Hydrogen bonds with FA carboxylate group atoms as acceptors					
H*H*	Tyr128	O1	1.73	0.31 (3)	1.8 (2)
H*E*	Arg126	O1	1.85	0.229 (5)	2.03 (2)
H2	*W*24	O2	1.86	0.25 (2)	1.62 (7)
H2	*W*13	O2	1.90	0.20 (2)	2.01 (5)
H*H*21	Arg126	O1	2.23	0.086 (5)	1.10 (7)
H*D*23	Leu115	O1	3.12	0.0168 (3)	0.283 (7)
C—H⋯O hydrogen bonds involving alkyl chain atoms					
O	*W*7	H62	2.55	0.050 (5)	0.82 (6)
O	*W*31	H181	2.55	0.054 (6)	0.7 (1)
O	*W*51	H51	2.63	0.049 (2)	0.80 (3)
O	Lys58	H183	2.82	0.033 (2)	0.56 (3)
O*D*1	Asp76	H82	2.86	0.026 (2)	0.36 (3)
O	Thr53	H182	2.98	0.023 (2)	0.26 (2)
O	*W*31	H31	3.38	0.0074 (5)	0.117 (6)
O	*W*31	H52	3.46	0.0057 (3)	0.101 (6)
O	*W*28	H141	3.47	0.006 (1)	0.09 (1)
