# Supplementary material for: High-resolution neutron and X-ray diffraction room-temperature studies of an H-FABP–oleic acid complex: study of the internal water cluster and ligand binding by a transferred multipolar electron-density distribution
Source: IUCrJ. 2016 Jan 16;3(Pt 2):115–26. doi: 10.1107/S2052252515024161 (PMC4775160; doi:10.1107/S2052252515024161)

IUCrJ

Volume 3 (2016)

Supporting information for article:

High resolution neutron and X-ray diffraction RT studies of an H-FABP – Oleic acid complex: study of the internal water cluster and the ligand binding by a transferred multipolar electron density distribution

E. I. Howard, B. Guillot, M. P. Blakeley, M. Haertlein, M. Moulin, A. Mitschler, A. Cousido-Siah, F. Fadel, W. M. Valsecchi, Takashi Tomizaki, T. Petrova, J. Claudot and A. Podjarny

Supplementary material

Text S1. Estimation of uncertainties on charge density derived properties.

In order to assess the significance of our results, we computed uncertainties on the reported properties following a statistical approach. First we assumed that errors on electrostatic and on AIM topological properties originate from uncertainties affecting atomic coordinates and transferred multipole parameters values. To account for these sources of errors, a total of 30 models were generated from the original H-FABP structure, where the atomic parameters (orthogonal coordinates, valence populations and non-zero multipole parameters) were randomly perturbed using Gaussian error functions. The standard deviations (±σ) associated to these normal distributions were adjusted to the errors estimated on the corresponding parameters. Here, errors on transferred (non-zero) multipole coefficients and atomic valence populations were taken as equal to the standard errors on the averaged values stored in the ELMAMII library, as distributed with the MoPro program suite. Errors on atomic coordinates were derived from Cruickshank diffraction precision index (DPI) formula (Cruickshank, 1999) using the "Online_DPI" web server (Kumar *et al.*, 2015). Using this method, errors on atomic coordinates are functions of isotropic B factors and of diffraction data and model quality. However, in our case, the perdeuterated H-FABP crystal structure was jointly refined against average resolution 1.9 Å neutron and 0.98 Å atomic resolution X-ray data sets. Hence, as the orientation of the water molecules and the position of hydrogen atoms (seen as deuterium) not determined by stereochemistry were provided by the neutron diffraction experiment, their coordinates errors were computed on the basis of the neutron data set (DPI = 0.29 Å), while those on all the other atoms were obtained using the high resolution X-ray data set (DPI = 0.03 Å). This choice resulted, as expected, on large uncertainties on hydroxyl group's and water molecule's hydrogen atoms, clearly reflecting uncertainties on their actual orientation (Figure S1). In a last step, on order to avoid including in the computations stereo chemically invalid models (as atomic coordinates perturbations were applied randomly and independently on each atoms), all the resulting 30 perturbed structures were subsequently regularized using standard stereo chemical restraints.

Finally, all electrostatic and QTAIM computations were re-performed for each of the 30 perturbed models. Hence, in this study, the electric field magnitudes, angles between electric field and water molecules dipole moment vectors, as well as electron density and Laplacian values on intermolecular bond critical points are given with standard errors on the mean values of properties obtained from these 30 independent computations, using a Student *t* distribution with 29 degrees of freedom (t = 2.75).

Table S1
Neutron data collection statistics at RT. 36 frames of 18.6 hrs each

	Overall	Inner Shell	Outer Shell
Low resolution limit (Å)	29.93	29.93	2.00
High resolution limit (Å)	1.90	6.01	1.90
Rmerge	**0.130**	0.094	0.200
Rmerge in top intensity bin	0.096	-	-
Total number of observations	59570	2153	4530
Total number unique	8714	392	1011
Mean((I)/sd(I))	**12.6**	14.9	7.3
Completeness (%)	**79.3**	96.3	65.0
Multiplicity	6.8	5.5	4.5

Table S2

X-ray data collection statistics at RT

	Overall	Inner Shell	Outer Shell
Low resolution limit (Å)	43.67	43.67	1.03
High resolution limit (Å)	0.98	3.10	0.98
Rmerge	0.056	0.045	0.892
Rmerge in top intensity bin	0.045	-	-
Rmeas (within I+/I-)	0.077	0.061	1.218
Rmeas (all I+ & I-)	0.072	0.057	1.196
Rpim (within I+/I-)	0.052	0.042	0.825
Rpim (all I+ & I-)	0.036	0.029	0.594
Fractional partial bias	0.000	0.000	0.000
Total number of observations	284204	9403	41091
Total number unique	73795	2581	10547
Mean((I)/sd(I))	6.8	18.2	1.1
Mn(I) half-set correlation CC(1/2)	0.996	0.994	0.546
Completeness (%)	93.5	94.6	92.6
Multiplicity	3.9	3.6	3.9
Anomalous completeness (%)	90.3	92.7	89.1
Anomalous multiplicity	2.1	2.1	2.1
DelAnom correlation between half-sets	-0.179	-0.167	-0.010
Mid-Slope of Anom Normal Probability	0.757		

Table S3

Refinement statistics X+N

Refinement	Neutrons	X-Rays
Resolution range (Å)	29.93-1.90	29.93-0.98
R_{factor}	0.21	0.14
R_{free}	0.25	0.16
No. of reflections for refinement	8710	73652
Model used for MR	1HMR	
$RMSD_{bonds}$ (Å)	0.014	
$RMSD_{angles}$ (°)	1.42	
Protein Atoms	1034	
Solvent molecules	189	
Ramachandran plot Favored (%)	98.5	
Ramachandran plot Accepted (%)	1.5	
Ramachandran plot Outliers (%)	0	

Table S4 Summary of all hydrogen bonds involving the 17 water molecules considered in the study. For clarity, all interactions of type OW-H…OW, where water molecules are acting simultaneously as donor and acceptor, are reported twice. Water molecules marked with asterisk are buried in the protein but are not located in the water cluster which fills the binding pocket along with the FA: W1 is the resident water conserved among nine different members of the FABP protein family (Likic *et al.*, 2000). W28 is in contact with the FA, but located on the other side than the water cluster; W38 is visible at the entrance of the water channel linking the hydration shell and the binding cavity.

	**Hydrogen bonds with water molecule as donor : OW-H…A**				**Hydrogen bonds with water molecule as acceptor : D-H…OW**			
**W**	H…A<br>Acceptor<br>atom	Distance<br>OW…A<br>(Å)	Distance<br>OW-H…A<br>(Å)	Angle<br>OW-H…A<br>(°)	D-H<br>Donor<br>group	Distance<br>D…OW (Å)	Distance<br>D-H…OW<br>(Å)	Angle<br>D-H…OW<br>(°)
**1***	H1…68 O<br>H2…65 O	2.802<br>2.810	2.033<br>2.071	153.5<br>146.8	84 N-H0	2.849	2.010	166.1
**3**	H1…104 O<br>H2…91 O	2.898<br>2.853	2.089<br>2.105	161.6<br>148.2	106 NH2-<br>HH11	2.905	2.142	148.5
**6**	H1…26 OW<br>H2…51 O	2.635<br>2.968	1.970<br>2.177	135.4<br>156.4	53 Oγ1-Hγ1<br>60 Oγ1-Hγ1	2.951<br>2.744	2.114<br>2.061	173.8<br>139.5
**7**	H1…19 OH<br>H2…95 OE1	3.028<br>2.784	2.214<br>2.010	162.8<br>152.7	51 OW-H2<br>78 NH2-HH22<br>133 C6-H62	3.047<br>2.898<br>3.287	2.440<br>2.078<br>2.548	129.8<br>159.3<br>124.3
**8**	H1…17 OW<br>H2…31 OW	2.775<br>2.773	2.215<br>2.310	124.3<br>115.2	51 OW-H1<br>75 N-H0	2.918<br>2.935	2.302<br>2.170	130.0<br>148.1
**13**	H1…20 OW<br>H2…133 O2	2.689<br>2.675	1.969<br>1.988	143.1<br>138.5	106 NH2-<br>HH21<br>40 OG1-HG1	2.920<br>2.723	2.073<br>1.962	167.5<br>150.6
**17**	H1…72 Oε2<br>H2…38 OW	2.675<br>2.850	1.989<br>2.312	138.3<br>122.4	8 OW-H1<br>26 OW-H2<br>74 Cα-Hα<br>60 Cβ-Hβ	2.775<br>3.086<br>3.864<br>3.916	2.215<br>2.257<br>2.898<br>3.209	124.3<br>169.1<br>173.8<br>131.1
**20**	H2…128 Cε1π<br>H2…128 Cδ1π	2.967<br>2.850	3.543<br>3.519	140.9<br>125.5	13 OW-H1<br>106 N-H0	2.689<br>2.831	1.969<br>1.979	143.1<br>170.9
**24**	H1…53 Oγ1<br>H2…133 O2	2.830<br>2.752	2.219<br>1.969	129.6<br>154.6	67A OW-H2<br>31 OW-H1	2.877<br>2.919	2.447<br>2.466	112.6<br>115.3
**26**	H1…67A OW<br>H1…103 OW<br>H2…17 OW	2.915<br>3.026<br>3.086	2.204<br>2.312<br>2.257	142.2<br>143.1<br>169.1	6 OW-H1<br>62 Cγ-Hγ12	2.635<br>3.834	1.970<br>3.109	135.4<br>132.7
**28***	H1…33 O<br>H2…36 O	2.826<br>2.728	2.136<br>2.083	139.3<br>133.2	126 NH1-<br>HH12	2.821	2.000	161.1
**30**	H2…103 OW	3.524	2.953	127.0	106 NH1-<br>HH12<br>93 Nδ1-Hδ1	2.732<br>2.743	1.894<br>1.938	166.1<br>155.5
**31**	H1…24 OW<br>H2…60 Oγ1	2.919<br>2.968	2.466<br>2.454	115.3<br>114.8	8 OW-H2<br>133 C18-<br>H181	2.773<br>3.585	2.310<br>2.516	115.2<br>166.1
**38***	H1…32 OW<br>H2…73 O	2.826<br>2.761	2.025<br>2.030	158.9<br>143.8	17 OW-H2<br>19 OW-H1<br>60 Cβ-Hβ	2.850<br>2.894<br>3.857	2.312<br>2.109<br>2.976	122.4<br>155.5<br>151.6

**51**	H1…8 OW<br>H2…7 OW	2.918<br>3.047	2.302<br>2.440	130.0<br>129.8	95 Nε2-Hε22<br>103 OW-H1	3.008<br>2.482	2.149<br>2.090	170.4<br>108.0
**67**	H2…24 OW	2.877	2.447	112.6	26 OW-H1<br>104 Cδ2-Hδ22<br>106 NH2-HH22	2.915<br>3.636<br>3.340	2.204<br>2.982<br>2.510	142.2<br>125.8<br>162.5
**103**	H2…72 Oε2<br>H1…51 OW	2.706<br>2.482	2.148<br>2.090	123.7<br>108.0	26 OW-H1<br>93 Cε1-Hε1<br>30 OW-H2	3.026<br>3.350<br>3.524	2.312<br>2.684<br>2.953	143.1<br>129.2<br>127.0

Figure S1

Superimposition of the 30 perturbed structures, represented in the region of the water cluster. The uncertainty on the water molecule’s orientations is seen through the continuum of acceptable orientations, arising from the neutron data resolution and the atomic B factors. The fatty acid can be seen at the bottom of the picture.

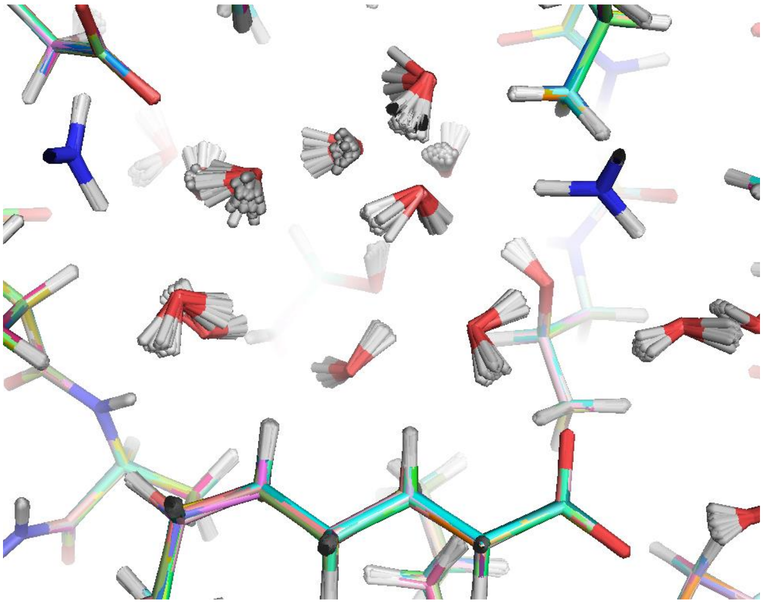

Figure S2

Relation between electric field magnitudes at water molecules center of mass and the raw angle measured between the electric field vector and the water molecule's dipole moments. Error bars correspond to standard error on the mean associated to these values (see materials and methods).

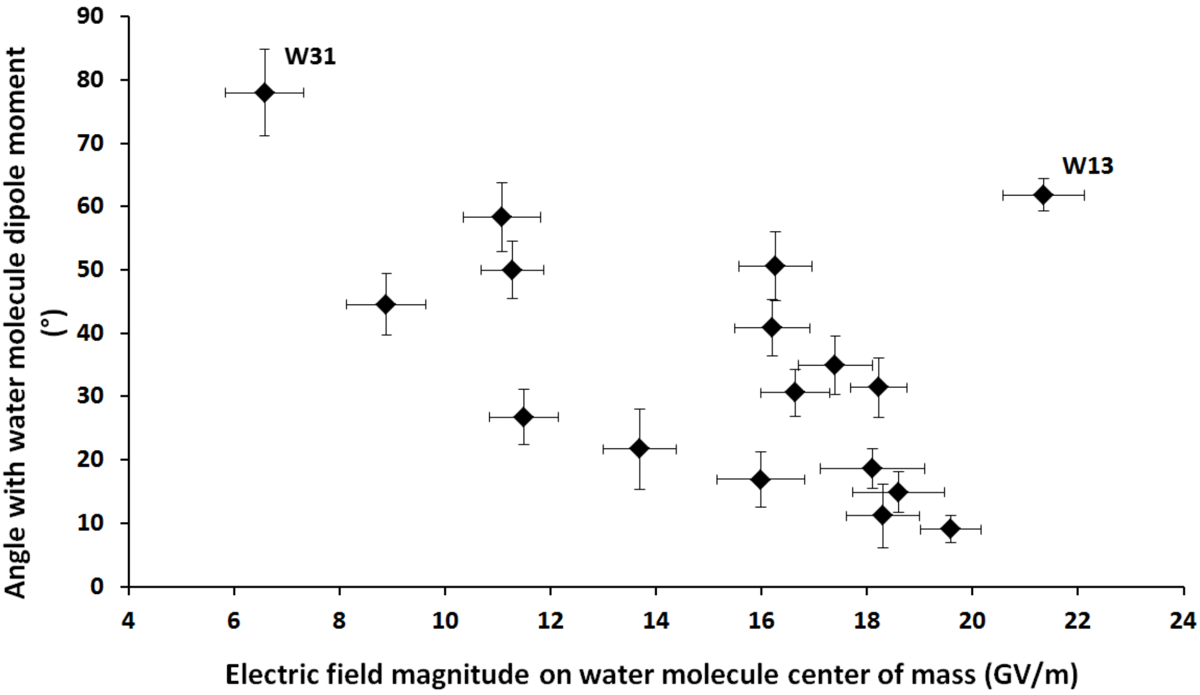

Figure S3

Superimposition of A-FABP apo form (PDB code: 3RZY, green waters and molecule) and the present structure showing that the water cluster molecules keep their position**.**

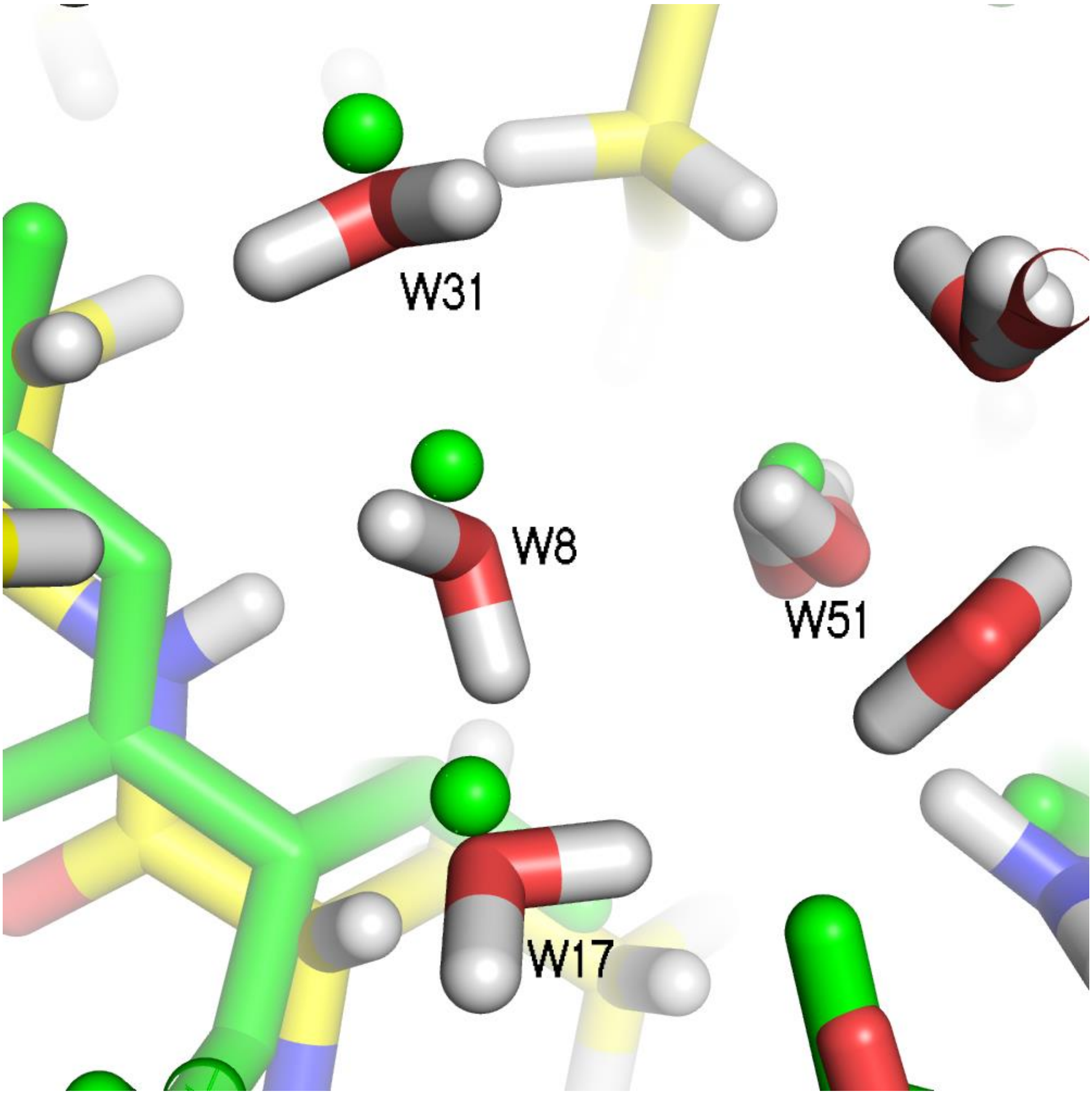

Supplement: Supplementary file 1 [file m-03-00115-sup1.pdf]
